# Early Initiation of Temozolomide Therapy May Improve Response in Aggressive Pituitary Adenomas

**DOI:** 10.3389/fendo.2021.774686

**Published:** 2021-12-17

**Authors:** Liza Das, Nidhi Gupta, Pinaki Dutta, Rama Walia, Kim Vaiphei, Ashutosh Rai, Bishan Dass Radotra, Kirti Gupta, Sreejesh Sreedharanunni, Chirag Kamal Ahuja, Anil Bhansali, Manjul Tripathi, Ridhi Sood, Sivashanmugam Dhandapani

**Affiliations:** ^1^ Department of Endocrinology, Postgraduate Institute of Medical Education and Research (PGIMER), Chandigarh, India; ^2^ Department of Histopathology, PGIMER, Chandigarh, India; ^3^ Department of Hematology, PGIMER, Chandigarh, India; ^4^ Department of Radiology, PGIMER, Chandigarh, India; ^5^ Department of Neurosurgery, PGIMER, Chandigarh, India

**Keywords:** temozolomide, aggressive pituitary adenomas, MGMT, controlled disease, early therapy

## Abstract

**Introduction:**

Aggressive pituitary adenomas (APAs) are, by definition, resistant to optimal multimodality therapy. The challenge lies in their early recognition and timely management. Temozolomide is increasingly being used in patients with APAs, but evidence supporting a favorable response with early initiation is lacking.

**Methods:**

This was a single-center study of all patients with APAs who received at least 3 cycles of temozolomide (150–200 mg/m^2^). Their baseline clinico-biochemical and radiological profiles were recorded. Immunohistochemical evaluation for cell-cycle markers O^6^-methylguanine-DNA methyltransferase (MGMT), MutS homolog 2 (MSH2), MutS homolog 6 (MSH6), MutL homolog 1 (MLH1), and postmeiotic segregation increased 2 (PMS2) was performed, and *h*-scores (product of the number of positive cells and staining intensity) were calculated. Response was assessed in terms of radiological response using the RECIST criteria. Patients with controlled disease (≥30% reduction in tumor volume) were classified as responders.

**Results:**

The study comprised 35 patients (48.6% acromegaly, 37.1% prolactinomas, and 14.3% non-functioning pituitary adenomas). The median number of temozolomide (TMZ) cycles was 9 (IQR 6–14). Responders constituted 68.6% of the cohort and were more likely to have functional tumors, a lower percentage of MGMT-positive staining cells, and lower MGMT *h*-scores. There was a significantly longer lag period in the initiation of TMZ therapy in non-responders as compared with responders (median 36 *vs*. 15 months, *p* = 0.01). ROC-derived cutoffs of 31 months for the duration between diagnosis and TMZ initiation, low-to-intermediate MGMT positivity (40% tumor cells), and MGMT *h*-score of 80 all had a sensitivity exceeding 80% and a specificity exceeding 70% to predict response.

**Conclusion:**

Early initiation of TMZ therapy, functional tumors, and low MGMT *h*-score predict a favorable response to TMZ in APAs.

## Introduction

Pituitary adenomas are predominantly benign tumors, with discernable dural, bony, or mucosal invasion in up to 35%–55% of patients (1). However, in neuroendocrine oncology especially pituitary oncology, locally invasive pituitary adenomas are not considered malignant neoplasms, unlike in general oncology (2). Aggressive pituitary adenoma (APA) refers to a tumor that is both invasive and proliferative or displays clinically meaningful tumor growth/recurrence despite optimal standard therapy (3, 4). The prevalence of APAs is nearly 10% as per current reports. Pituitary carcinomas (PCs) are exceedingly rare with a prevalence of 0.2% (5). Despite the parlance, the clinical behavior, proliferative markers, resilience to multimodality therapy, and outcomes of APAs and PCs are very similar (6). Unlike other neuroendocrine neoplasms which are stratified by the WHO grade and TNM stage, pituitary adenomas have been classified by the recent clinicopathological grade which incorporates both invasion (basis of the stage) and proliferation (basis of grade) (7).

Of late, management with temozolomide (TMZ), an oral alkylating agent, has been attempted substantially and endorsed as the first-line chemotherapeutic agent for APAs and PCs (4). Though initially used as war weapons, alkylating agents found their way in neuro-oncology nearly four decades back with FDA approval for their use in glioblastoma multiforme (8). The first use of TMZ in APAs dates back to 2004 where it was reported by Lim et al. (9). Temozolomide, by virtue of its small molecular size, readily crosses the blood–brain barrier. Its efficacy is due to its non-cell-cycle-specific nature, thereby facilitating its use in slowly growing tumors such as pituitary adenomas. It is the only chemotherapeutic agent with established efficacy in treating APAs, with success in improving both progression-free survival (PFS) and overall survival (OS) (10–12).

The current study aims to present our experience of managing APAs with temozolomide therapy and analyze the clinico-radiologic and hormonal outcomes of these patients as well as determinants of these outcomes.

## Patients and Methods

This is a single-center, prospective study (2006–2021) performed at the multidisciplinary pituitary clinic in the Department of Endocrinology, PGIMER, Chandigarh. The study was approved by the Institutional Ethics Committee (INT/IEC/2021/SPL-990) and written informed consent was obtained from all the patients. The criteria for classifying a pituitary adenoma as aggressive in the current study included multidisciplinary consensus definition on the basis of dural invasion (radiological/histopathological/intraoperative) or proliferation (commonly measured by Ki67 >3%), as per the workflow in **Supplementary Figure 1**.

Only those who received at least 3 cycles of temozolomide were included in the study. Their demographic, clinical, biochemical, hormonal, and radiological profiles (*n* = 35) were studied. All the patients were followed prospectively, either from the treatment naive stage or before starting TMZ till the last follow-up.

### Baseline Assessment

All hormones were assessed at baseline, before starting TMZ, and during follow-up, using electrochemiluminescence (ECLIA) (eCOBAS 601, Roche, Hitachi), except IGF-I, which was measured by the DiaSorin-Liaison assay. The intra-assay coefficient of variation (CV) was 1.8%–4.1% and the inter-assay CV was 2.7%–5.7% for ECLIA, and the intra-assay CV was 4.5% and the inter-assay CV was 4.3% for the DiaSorin-Liaison assay. Hormones were analyzed in absolute values and expressed as times the upper limit of normal to derive hormone indices (absolute value/upper limit of normal for that age and gender) as unitless numbers. Magnetic resonance imaging (MRI) sella was performed on a 3-T MRI machine, with 1 mm thickness slices. The dimensions were observed by a single expert radiologist (CA). Adenoma volume was calculated by the ellipsoid formula [volume = (sagittal × coronal × axial diameters) × π/6]. Serial MRIs were performed to assess the tumor volume.

### Temozolomide Treatment Regime and Response Criteria

TMZ was used in the form of oral preparation (capsules) available in various strengths, such as 20, 100, and 250 mg. All patients were administered TMZ in a standard dose of 150 mg/m^2^ body surface area (BSA) in the first cycle and 200 mg/m^2^ for subsequent cycles, in a 5/28 regimen. The dose obtained after calculation was rounded off to the nearest possible strength. Cumulative dose was calculated as the total dose of TMZ received by a given patient (sum of doses administered during each cycle). Response to therapy was assessed both in terms of radiological and hormonal response (only in functioning tumors) individually and in a combined manner. Both radiological and hormonal responses were defined from the time of TMZ initiation till its discontinuation. The response evaluation criteria in solid tumors (RECIST) criteria were used to identify patients with controlled disease (CD) [either complete response (CR) defined as the disappearance of all lesions or partial response (PR) defined in patients with at least 30% reduction in tumor volume], stable disease (SD) (change in tumor volume between 20% decrease and 10% increase from baseline), or progressive disease (PD) (≥20% increase in the tumor volume). The combined overall (radiological and hormonal) response was defined as the presence of normalization of hormone levels after discontinuation of TMZ along with at least a 30% reduction in tumor volume. In non-functioning pituitary adenomas (NFPAs), reduction of tumor volume alone was defined as a complete response.

### Histopathology

Histopathological examination (HPE) was performed on biopsy/tissue specimens of all operated patients. HPE evaluation was performed by two pathologists (KG, BR) at a dedicated neuropathology unit. For patients operated on more than once, tumor tissue from the latest surgery before initiating TMZ therapy was considered for the study. Immunohistochemistry (IHC) for all six anterior pituitary hormones, i.e., growth hormone (GH), prolactin (PRL), luteinizing hormone (LH), follicle-stimulating hormone (FSH), adrenocorticotrophic hormone (ACTH), and thyroid-stimulating hormone (TSH), was performed. Ki67, p53, and mitotic index were performed on all operated specimens.

### Immunohistochemistry and Tumor Markers

Paraffin-embedded tissue sections were deparaffinized and antigen retrieval was carried out using citrate buffer (pH = 6.0). Hydrogen peroxide was used for quenching the endogenous peroxide. IHC for O^6^-methylguanine-DNA methyltransferase (MGMT), MutS homolog 6 (MSH6), MutS homolog 2 (MSH2), MutL homolog 1 (MLH1), and postmeiotic segregation increased 2 (PMS2) was carried out on 21 tissue specimens of 29 operated patients. The tissue sections were incubated with primary antibodies for MGMT (MT 3.1, 1:100, Dako), MSH6 (44, 1:100, Bio SB, USA), MSH2 (G-219-1192, 1:100, Bio SB, USA), MLH1 (ab 92312, 1:100, Abcam, UK), and PMS2 (ab 110638, 1:100, Abcam, UK). After washing, slides were incubated with horseradish peroxidase (HRP)-conjugated secondary antibodies and signal was developed using DAB (Lab Vision™ UltraVision™ ONE Detection System). For all IHC parameters, each specimen was assessed for percentage of positively staining cells and the intensity assessment was performed as follows: 0 = negative, 1 = low positive, 2 = medium positive, and 3 = strong positive. The intensity of staining and the number of stained cells were considered for each case and the product was calculated to obtain the *h*-score. Positive controls used for immunohistochemistry were tissue specimens of colon cancer, and negative controls were evaluated by omission of primary antibody in the tissue specimens.

### Statistical Analysis

Statistical analysis was performed using the Statistical Package for Social Sciences (SPSS) 22.0 software program (IBM Statistics 22.0). Quantitative parameters were checked for normality using the Kolmogorov–Smirnov method and classified as parametric or non-parametric. Qualitative variables were compared between the groups using Pearson *χ* ^2^ or Fisher’s exact test. Student’s *t*-test was used to compare the means of two groups for parametric and Mann–Whitney *U* test for non-parametric data. Continuous variables were compared between groups using Wilcoxon matched-pair test. *p*-values less than 0.05 were considered significant. Data are presented as *n* (%), median (quartile q25–q75), or mean ± SD.

## Results

### Baseline Demographics

The study comprised a total of 35 patients, including 48.6% (*n* = 17) patients with acromegaly, 37.1% (*n* = 13) with prolactinomas, and 14.3% (*n* = 5) with NFPAs. Compressive features (82.8%) were more common than features of hormonal hypersecretion (66.7%). The most common secondary dysfunction was hypogonadism (83.3%) followed by hypocortisolism (68.7%) and hypothyroidism (56.2%). All patients had macroadenomas and 51.7% had giant adenomas. MRI evidence of invasion (defined as Knosp grades 3 and 4) was present in 88.9% of patients. The workflow for defining aggressive pituitary adenomas and the choice of TMZ therapy is outlined in **Supplementary Figure 1**.

### Course of TMZ Therapy

All patients with NFPA and 94.1% of patients with acromegaly underwent primary surgery. One patient with acromegaly underwent primary radiotherapy (intensity-modulated radiotherapy, IMRT) followed by long-acting depot preparation of octreotide monthly with dose escalation from 20 to 60 mg. However, due to non-responsiveness, she was initiated on temozolomide, which resulted in SD following 16 months of therapy. Among prolactinomas, 53.8% underwent surgery, all following failed response to optimal doses of cabergoline. All patients were resistant to either one or multiple courses of medical/radiation therapy and surgeries. Of these, 94.3% were invasive (MRI or HPE), 31.4% were proliferative (Ki67 >3%, mitotic index >2/10 high power fields or p53 positivity), and 28.6% were both invasive and proliferative. Two patients with giant prolactinomas who had good hormone response (normalization) with cabergoline but failed tumor size reduction were managed with TMZ, after being found non-responsive to other modalities of management such as surgery, radiotherapy (RT), or gamma knife radiosurgery (GKRS). Multiple surgeries (≥2) were required in six patients (two patients with acromegaly, one with NFPA, and three with prolactinoma). Among those who received radiotherapy (60%), multiple sessions of radiotherapy were required in five patients (four patients with acromegaly and one with prolactinoma). GKRS was administered to 30.7% and IMRT/EBRT to 69.3%. The minimum duration between two sessions of RT was 3 years, and only one patient received GKRS on both occasions, whereas the others received a combination of IMRT and GKRS.

Complete HPE data were available in 72.4% (*n* = 21) patients. Dural, sphenoid mucosal, or bony invasion was present in 79% of patients. Standard monthly dosing of TMZ of 150–200 mg/m^2^ BSA was used every 5/28 days. The median age of the cohort at diagnosis was 34.5 (26.5–40) years and the median age at initiation of treatment with TMZ was 38 (29.5–41) years. TMZ was initiated at a median of 20 (14–36.5) months from the time of diagnosis and administered for 9 (6–14) cycles.

**Figure 1:**
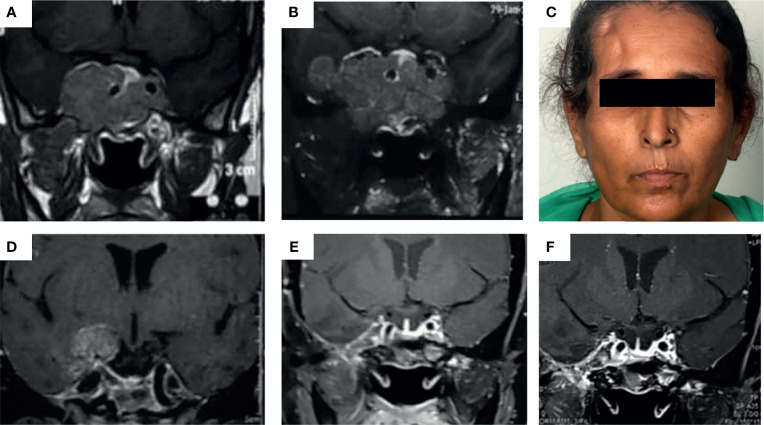
**(A–E)** Images depicting the baseline MRI of a patient presenting with headache and secondary amenorrhea due to an invasive macroprolactinoma **(A)** which was dopamine agonist resistant. She underwent transsphenoidal surgery elsewhere with significant tumor residue **(B)**, necessitating redo surgery by the transfrontal route at our institute (right pterional craniotomy) **(C)**. Due to persistent right temporal lobe residual mass **(D)**, the patient was managed with intensity-modulated radiotherapy (50 Gy over 28#) along with initiation of temozolomide therapy at 150 mg/m^2^ dose for the first month followed by 200 mg/m^2^ dose in 5/28 day cycles, for a total of 9 cycles. This led to almost complete resolution of the tumor in the patient as well as normalization of serum prolactin **(E)** which was persistent even after 2 years of follow-up **(F)**.

TMZ was most commonly used as the third line of management (in 48.6% of patients). The time for TMZ use was based on multiple factors such as established or anticipated aggressiveness, resistance, non-affordability or intolerance to standard drugs (somatostatin receptor ligands or dopamine agonists), and multidisciplinary consensus. The common reasons for early institution of therapy were dopamine agonist (DA) resistance in prolactinomas, non-affordability [cost of therapy with TMZ is nearly one-third that of somatostatin receptor ligands (SRLs) in our settings], and non-willingness for injectable SRLs as well as for interim medical therapy following radiotherapy in acromegaly and non-functioning pituitary adenomas. It was used concurrently with other therapies in certain patients. In a boy with childhood aggressive somatotropinoma due to AIP mutation, TMZ was used along with bevacizumab for a synergistic effect (13). For prolactinomas, two patients showed normalization of hormone levels with DA, but with significant residual tumor causing compressive effects, which was managed successfully with TMZ. A 24-year-old male with a giant prolactinoma who developed CSF rhinorrhea with DA underwent transsphenoidal surgery (TSS) and was managed with TMZ for a huge residual mass, leading to complete response. In another patient who developed impulse control disorder (hypersexuality) on DA, TMZ was offered following failed TSS (with concurrent RT). However, he failed to respond, prompting redo surgery and HPE revealed a high Ki67 index and 100% MGMT positivity. Another patient who presented with an aggressive, invasive macroprolactinoma with residual disease after TSS followed by transfrontal surgery was managed with TMZ (9 cycles) and radiotherapy, leading to her complete response (**Figure 1**).

### Response to TMZ

The overall median hormone reduction in the cohort was 42.5% (12%–75%) and tumor volume reduction was 54% (21.5%–98%). Significant radiological response (CD) was seen in 66.7% and normalization of hormone was seen in 51.6% of patients. Using the RECIST criteria, patients with controlled disease (complete or partial response) were classified as responders and they constituted 68.6% of the cohort. Of these, 40.9% had complete response and 59.1% had partial response. Non-responders referred to both stable disease (change in tumor volume between 20% decrease and 10% increase from baseline) and progressive disease (≥20% increase in the tumor volume). Of the non-responders, SD constituted 72.7% and progressive disease was seen in 27.3% of patients. Their baseline characteristics are summarized in **Tables 1**–**3**. Functional tumors constituted significantly greater proportion of responders (95.8%) as compared with non-responders (63.6%) (*p* = 0.01). Radiological invasion was present in all non-responders and most responders (87.5%) (*p* = 0.22). However, on HPE, invasion (bone, dura, and mucosa) was present in all patients. None of the other histopathological parameters (Ki67, mitotic index) were significantly between both subgroups. Among the patients with functional tumors that did not respond by reduction in adenoma volume (*n* = 7), five had decreased hormone levels, with a median reduction of 50% (IQR 26–96.5) from the baseline. Two patients (one each with acromegaly and one with prolactinoma) did not have any reduction in hormone levels from baseline besides not responding significantly in terms of adenoma volume. There was no significant difference based on the subtype of tumor (**Supplementary Table 1**). Details of the immunohistochemical analysis for hormones and transcription factors in case of NFPAs are listed in **Supplementary Table 2**.

**Table 1:** Baseline demographic and clinical, radiological, and histopathological parameters in the cohort of patients managed with temozolomide.

Parameter	Non-responder (*n* = 11)	Responder (*n* = 24)	*p*-value
Age at diagnosis (years)	38 ± 9.39	31.7 ± 13.8	0.13
Female sex, % (*n*)	45.5 (5)	50 (12)	0.80
Functioning pituitary adenomas, % (*n*)	**63.6 (7)**	**95.8 (23)**	**0.01**
Type of tumor, % (*n*)			
Acromegaly	**36.4 (4)**	**54.2 (13)**	**0.04**
NFPA	**36.4 (4)**	**4.2 (1)**	
Prolactinoma	**27.3** (3)	**41.6 (10)**	
Compressive features, % (*n*)	72.7 (8)	83.3 (20)	0.46
Symptoms of hormonal hypersecretion, % (*n*)	85.7 (6)	63.6 (14)	0.27
Baseline hypothyroidism, % (*n*)	50 (5)	61.9 (13)	0.53
Baseline hypocortisolism, % (*n*)	66.7 (6)	72.7 (16)	0.73
Baseline hypogonadism, % (*n*)	77.8 (7)	90 (18)	0.37
Apoplexy, % (*n*)	27.3 (3)	20.8 (5)	0.67
Baseline adenoma volume (mm^3^)	15,830 (3,388–24,198)	13,056 (6,048–34,560)	0.86
Giant adenomas, % (*n*)	50 (5)	42.9 (9)	0.73
Invasion on MRI, % (*n*)	100 (11)	87.5 (21)	0.22
Baseline hormone value (ULN)^a^	3.5 (3.2–224.3)	5 (3.2–134)	0.64
Invasion on HPE, % (*n*)	83.3 (5)	83.3 (10)	1.00
Proliferative, % (*n*)	36.4 (4)	29.2 (7)	0.67
Ki67 (%)	2 (1–8)	1 (1–3)	0.68
Trouillas’ grading, % (*n*)			
Grade 2a	63.6 (7)	70.8 (17)	0.29
Grade 2b	36.4 (4)	29.2 (7)	
Mitotic index	2 (0–3.25)	3 (0.75–4.0)	0.66
Invasive and proliferative, % (*n*)	27.3 (3)	29.2 (7)	0.90

Data are expressed in % (n), mean ± SD, or median (q25–q75), as appropriate.

NFPA, non-functioning pituitary adenomas; MRI, magnetic resonance imaging; ULN, upper limit of normal.

a Only for functioning pituitary adenomas.

Bold values denote parameters that were significantly different between responders and non-responders.

**Table 2:** Comparative parameters between responders and non-responders in terms of the type and timeline of interventions used in the cohort.

Parameter	Non-responder	Responder	*p*-value
Age at first intervention (years)	37.9 ± 8.6	31.7 ± 13.8	0.12
Type of first intervention, % (*n*)			
Surgery	54.5 (6)	60.9 (14)	0.34
RT	9.1 (1)	0 (0)	
Drug	36.4 (4)	39.1 (9)	
Age at second intervention (years)	40.1 ± 11.0	32.8 ± 13.4	0.10
Type of second intervention, % (*n*)			
Surgery	45.4	23.1	0.11
RT	27.3	61.5	
Drug	27.3	15.4	
Age at third intervention (years)	**41.2 ± 9.9**	**32.0 ± 13.7**	**0.05**
Type of third intervention, % (*n*)			
Surgery	–	–	0.30
RT	36.4 (4)	18.8 (3)	
Drug	63.6 (7)	81.2 (13)	
Age at fourth intervention (years)	43.6 ± 5.1	30.8 ± 17.4	0.12
Type of fourth intervention, % (*n*)			
Surgery	–	–	0.43
RT	0 (0)	11.1 (1)	
Drug	100 (5)	88.9 (8)	

Data are expressed in % (n) or mean ± SD, as appropriate.

RT, radiotherapy.

Bold values denote parameters that were significantly different between responders and non-responders.

**Table 3:** Comparative parameters between responders and non-responders with respect to course and outcomes of temozolomide therapy.

Parameter	Non-responder	Responder	*p*-value
Age at TMZ initiation (years)	40 (36.5–46.5)	34 (27.2–41)	0.09
Duration of TMZ use (number of cycles)	6 (3–15.2)	10 (6–13.5)	0.10
Cumulative TMZ dose (mg)	11,675 (7,912–18,282)	15,225 (9,900–29,162)	0.25
Adenoma volume prior to TMZ use (mm^3^)	8,363 (2,981–27,107)	6,647 (1,512–17,144)	0.55
Adenoma volume after TMZ use (mm^3^)	**7203 (2,816–42,960)**	**504 (56–3,952)**	**0.00**
Hormonal level before TMZ use (ULN)^a^	2.95 (2.09–188)	2.24 (0.99–3.56)	0.08
Hormonal level after TMZ use (ULN)^a^	**3.32 (1.24–14.75)**	**0.86 (0.32–1.80)**	**0.02**
TMZ number in the treatment sequence		
2	9.1	33.3	0.31
3	54.5	45.8	
4	36.4	16.7	
	0	4.2	
Duration between diagnosis and TMZ use (months)	**36 (17.25–74)**	**15 (12–25.5)**	**0.01**
TMZ with RT, % (*n*)	54.5 (6)	43.5 (10)	0.54
Adverse effects, % (*n*)	55.6 (5)	47.6 (10)	0.69
Operated, % (*n*)	100 (11)	75 (18)	0.06
Multiple surgeries, % (*n*)	**40** (4)	**8.3** (2)	**0.02**
RT, % (*n*)			
0	36.4 (4)	41.7 (10)	0.89
Once	45.5 (5)	45.8 (11)	
More than once	18.2 (2)	12.5 (3)	
Proportion of adenomas with hormonal normalization, % (*n*)	**14.3** (1)	**65.2 (15)**	**0.01**
Hormone response on TMZ	40 (0–75)	44 (20–75)	0.71
Tumor response on TMZ	**7 (0–19.7)**	**72** (42–99)	**0.00**
Both normalization of hormone and a significant reduction in adenoma volume	**0** (11)	**66.7 (24)**	**0.00**
Hormone normalization at follow-up	40 (2)	80 (16)	0.07
Hormone response after stopping TMZ, % (*n*)	50 (10–61)	18.5 (0–43.5)	0.60
Tumor response after stopping TMZ, % (*n*)	11.5 (0–85.5)	5.9 (0–50.7)	0.97
Duration of follow-up from baseline (months)	78 (51–132)	60 (35.2–87)	0.30
RECIST response, % (*n*)			
CD	**0 (0)**	**95.7 (22)**	**0.00**
SD	**72.7 (8)**	**4.3 (1)**	
PD	**27.3 (3)**	**0 (0)**	

ULN, upper limit of normal; RT, radiotherapy; CD, controlled disease; SD, stable disease; PD, progressive disease.

a Only for functioning pituitary adenomas.

Bold values denote parameters that were significantly different between responders and non-responders.

Additional treatment besides TMZ was required by all patients with acromegaly and 80% of patients with NFPAs. TMZ was used as the second line of therapy in most patients (61.5%) with prolactinomas, following refractoriness to optimal doses of cabergoline. For the rest of the patients, it was used as the third or fourth line of therapy. Both multiple surgeries (*p* = 0.02) and multiple sessions of radiotherapy (*p* = 0.89) were required more frequently in non-responders. There was no significant difference between the age at which TMZ was initiated between both subgroups (*p* = 0.09). However, there was a significantly longer lag period in the initiation of TMZ therapy in the case of non-responders as compared to responders (median 36 *vs*. 15 months, *p* = 0.01). Responders received a median of 10 (6–13.5) cycles of TMZ as compared with non-responders who received 6 (3–15.2) cycles (*p* = 0.10). There was no significant difference between the adenoma volumes or hormone levels at baseline between responders and non-responders, but both parameters were significantly lower after TMZ use in the responders. The median reduction in hormone levels was not significantly different between both subgroups, but the median reduction in tumor volume was significantly higher in responders (*p* = 0.00). Concurrent chemoradiotherapy (TMZ with radiotherapy) was used in a similar proportion of patients in both subgroups (*p* = 0.54). Both normalization of hormone and CD was present in two-thirds of responders and none in the non-responders (*p* = 0.00). Progressive disease was present in 14.3% of non-responders and none of the responders. Individual patient response in terms of hormonal and tumoral response to TMZ is depicted in **Supplementary Figure 2**.

### Histopathology and Immunohistochemistry Evaluation

IHC of various cell-cycle markers was performed and *h*-scores were derived for each parameter (**Table 4**). There was a significantly higher number of cells staining positive for MGMT among non-responders (median 50% *vs*. 0%, *p* = 0.03). A trend toward significance was noted in terms of absent-to-low staining for MGMT among responders (*p* = 0.09). The median *h*-score for MGMT was significantly lower among responders. There was no significant difference between both subgroups in terms of the number of positive cells and intensity for the other markers, namely, MSH2, MLH1, and PMS2. In terms of MSH6, there was a significantly higher proportion of positively staining cells among responders (median 50% *vs*. 20%, *p* = 0.05).

**Table 4:** Comparative histopathological and immunohistochemical parameters between responders and non-responders.

Parameter	Non-responder	Responder	*p*-value
MGMT (%) cells	**50 (10–100)**	**0 (0–15)**	**0.03**
MGMT staining intensity			
0	14.3	57.1	0.09
Low	0	14.3	
Moderate	57.1	14.3	
Strong	28.6	14.3	
MGMT *h*-score	**200 (20–240)**	**0** (0–30)	**0.02**
MSH2 (%) cells	0 (0–20)	0 (0–4)	0.96
MSH2 staining intensity			
0	71.4	61.5	0.35
Low	28.6	15.4	
Moderate	0	23.1	
Strong			
MSH2 *h*-score	0 (0–20)	0 (0–4.5)	0.88
MSH6 (%) cells	**20 (0–40)**	**50 (15-62.5)**	**0.05**
MSH6 staining intensity			
0	42.9	15.4	0.21
Low	14.3	0	
Moderate	28.6	46.2	
Strong	14.3	38.5	
MSH6 *h*-score	30 (0–80)	120 (30–187.5)	0.06
MLH1 (%) cells	30 (0–60)	40 (30–55)	0.39
MLH1 staining intensity			
0	28.6	0	0.16
Low	0	7.7	
Moderate	57.1	53.8	
Strong	14.3	38.5	
MLH1 *h*-score	60 (0–120)	80 (60–130)	0.35
PMS2 (%) cells	0 (0–40)	20 (13.5–35)	0.35
PMS2 staining intensity			
0	57.1	15.4	0.21
Low	0	7.7	
Moderate	28.6	30.8	
Strong	14.3	46.2	
PMS2 *h*-score	0 (0–80)	40 (30–70)	0.39

Data are expressed in % (n), mean ± SD, or median (q25–q75), as appropriate.

MGMT, methylguanine methyltransferase; MSH2, MSH6, MLH1, mismatch repair protein complex.

Bold values denote parameters that were significantly different between responders and non-responders.

### Adverse Events

**Figure 2:**
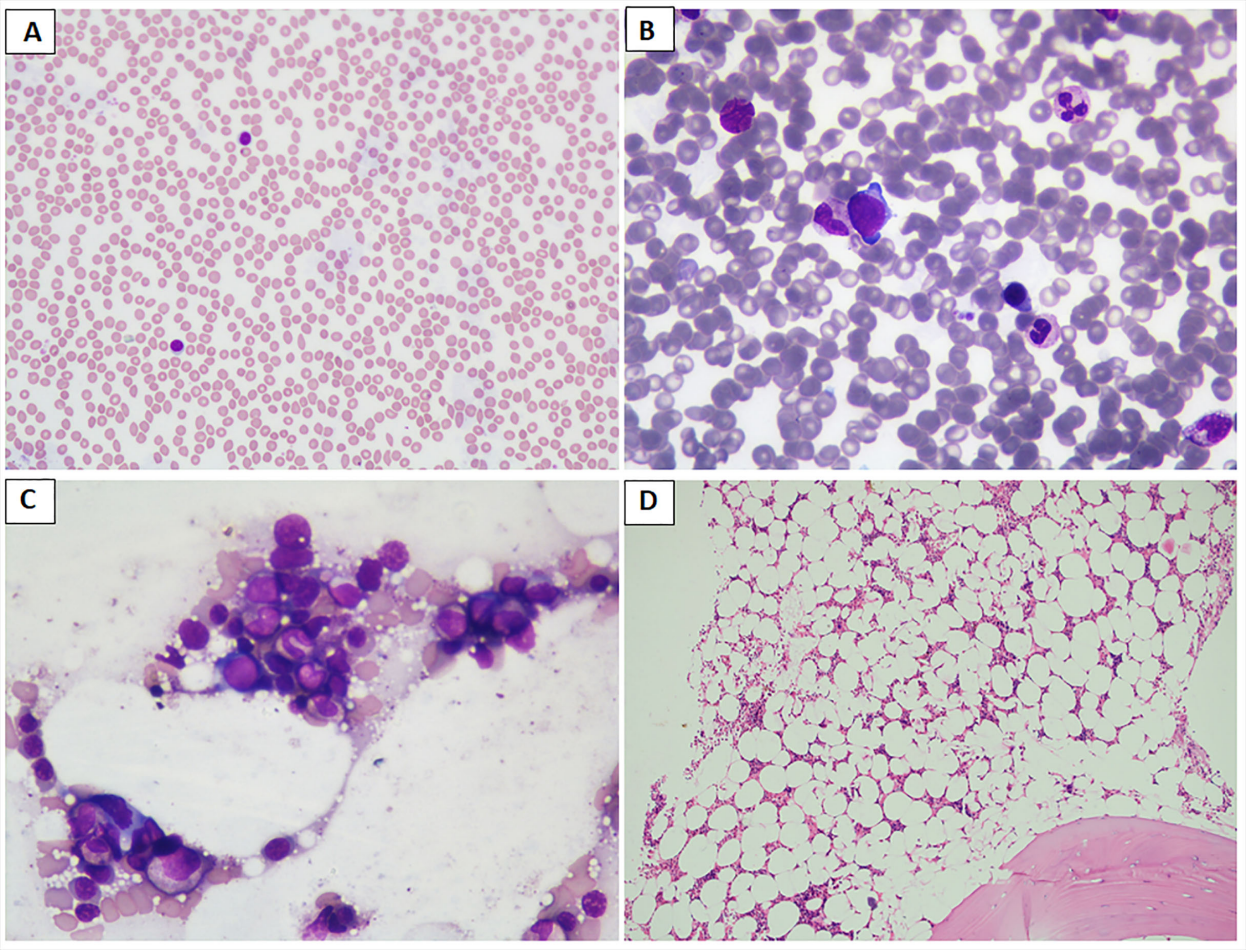
**(A–D)** Images depicting the bone marrow biopsy specimen of a patient who presented with acrogigantism due to an aggressive pituitary adenoma and subsequently developed bone marrow aplasia following temozolomide therapy, leading to discontinuation of the drug. **(A)** Peripheral blood film shows neutropenia and predominantly lymphocytes (May Grunwald–Giemsa stain/MGG; 20×); **(B, C)** paucicellular bone marrow aspirate and imprint smears show scattered myeloid and erythroid precursors (MGG; 40×); **(D)** trephine biopsy shows moderately to markedly hypocellular bone marrow spaces (hematoxylin and eosin stain; 10×). Later, the patient was reinitiated on long-acting octreotide therapy at a 30-mg monthly dose, leading to remission of disease activity, probably attributable to prior gamma knife radiosurgery.

TMZ was generally well tolerated, with a 50% prevalence of any adverse events (AEs) irrespective of severity (*p* > 0.05). The most common AEs were gastrointestinal (nausea, vomiting, constipation), fatigue, and weakness. Using the CTCAE grading, grade 1 or 2 AE was present in 67% of patients. Among those with severe adverse events, three patients had cytopenias and one had hyponatremic encephalopathy within 7 days of initiation of TMZ. Of those with cytopenias (all responders), one patient with acrogigantism developed anemia and thrombocytopenia, requiring stoppage of TMZ and initiation of SRL (**Figure 2**). The other two patients had pancytopenia, requiring discontinuation of TMZ albeit with 83% and 99.7% reduction of tumor volume from baseline. However, one of them required treatment with GM-CSF, transfusions with blood products, and antifungals to manage cytopenia. The patient with hyponatremia improved with hypertonic saline and hydrocortisone administration. However, the patient was restarted TMZ after a brief period of discontinuation, and this was continued uneventfully for 15 cycles without any recurrence of hyponatremia. Another patient with acromegaly had CSF rhinorrhea following TMZ, which was stopped and replaced by DA. None of the patients died due to cytopenia or any other complications.

### Long-Term Outcomes

The median duration of follow-up of the cohort was 60 (36–96) months. Following a maximal duration of TMZ use of 16 months in the non-responders and 60 months in the responders, the proportion of patients who normalized their hormone levels at follow-up was higher among responders (80%) than non-responders (40%) (*p* = 0.07). The duration of follow-up after TMZ use was a median of 6.5 months (IQR 1–51) among responders and 23 months (IQR 4–36) among non-responders. A significantly higher proportion of responders showed normalization of hormone levels following discontinuation of TMZ (65.2 *vs*. 14.3%, *p* = 0.01). There was one death in the cohort, a patient with DA-resistant, aggressive giant prolactinoma who showed progressive disease despite multimodality therapy. TMZ was initiated along with redo surgery and a second session of radiotherapy, but she had infratemporal spread of the disease with probable metastases (lymph node), multiple lower cranial nerve palsies, inanition, and death. There were no other instances of long-standing cytopenias or irreversible bone marrow suppression.

**Figure 3:**
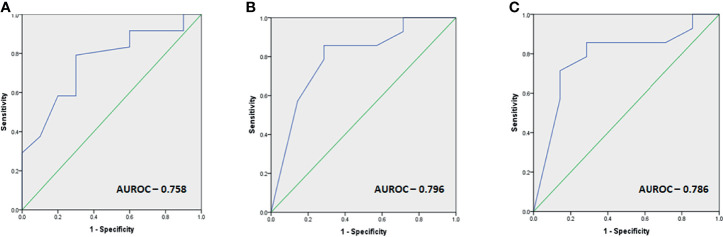
**(A–C)** Images depicting the ROC cutoffs for the various parameters found significant in response to TMZ with **(A)** ROC cutoff depicting the optimal duration before initiation of TMZ therapy as 31 months (80% sensitivity, 70% specificity), **(B)** optimal MGMT percentage of cells as 40% for predicting response (86% sensitivity, 72% specificity), and **(C)** optimal MGMT *h*-score as 80 (86% sensitivity, 72% specificity) for predicting response.

Receiver-operating curve (ROC) cutoffs of the parameters found significant for predicting response to TMZ are summarized in **Figure 3**.

## Discussion

The current study is one of the largest, single-center cohorts managed by a dedicated multidisciplinary pituitary team. It provided an opportunity to evaluate the efficacy and safety of temozolomide therapy in aggressive pituitary tumors in which it is lesser studied, such as somatotropinomas, prolactinomas, and NFPAs. We found a response rate (RR) of nearly 68%, with a greater reduction in tumor volume than hormone level. Functional tumors (acromegaly or prolactinomas) were found to have a higher likelihood of response than non-functional tumors. Responders were found to have a significantly earlier initiation of therapy as compared with non-responders. The percentage of MGMT-positive cells was significantly lower and those expressing MSH6 were significantly higher in responders than non-responders. ROC-derived cutoffs of 31 months of initiation of TMZ therapy, low-to-intermediate MGMT positivity (40% tumor cells), and MGMT *h*-score of 80 all had a sensitivity of at least 80% and a specificity of at least 70% to predict response. The current study endorses the findings of tumor functionality and low-to-intermediate MGMT as being associated with a favorable response and suggests that earlier initiation of TMZ in the management algorithm may be associated with better RR even when using stringent criteria to define disease control.

Pituitary adenomas are among the common intracranial neoplasms, and while most show benign behavior, around 20% have unfavorable surgical outcome with 10% requiring retreatment (14, 15). Clinicopathologically, 10% of adenomas are aggressive or rarely malignant (0.2%) (3). Though APAs have been variably defined as both invasive and proliferative (Trouillas 2b) or as tumors having clinically relevant tumor growth despite optimal multimodality therapy, the real challenge in the definition lies in the early identification of a tumor as being aggressive. The definition of APA itself entails a long lag period before the aggressiveness of a particular tumor is recognized, following failure of surgery, radiotherapy, and/or medical management (4). There are no well-defined baseline clinical, radiological, histopathological, or molecular markers of aggressiveness. This usually prohibits an earlier initiation of temozolomide in APAs. However, despite evidence that early initiation of TMZ prolongs overall survival in glioblastomas, similar data in APAs are lacking (16). The current study enabled the evaluation of the efficacy and safety of early initiation of TMZ in both functional and non-functional APAs, and we found that early initiation of TMZ (as early as second therapy in prolactinomas and third therapy in acromegaly and NFPAs) was associated with good response. In our study, all the tumors showed aggressive behavior, with a potential to invade locally, prolactinoma with but without any evidence of distant metastases consistent with carcinomatous transformation. Temozolomide use in these aggressive and invasive pituitary macroadenomas exhibited an expeditious response in hormone levels and tumor volume reduction. However, this response was observed maximally until patients were on TMZ, which continued to remain stable or declined after stopping TMZ. The legacy effect noted with a further significant reduction of tumor volume (*n* = 9) and normalization of hormone levels (*n* = 18) could not be attributed only to TMZ alone, as there was a coexisting effect of other therapies, such as radiation therapy, Sandostatin LAR, and dopamine agonists.

Ki67 is a well-established marker of proliferation, with a cutoff of 3% being the most commonly used. However, its controversial role has been acknowledged and lower thresholds of 2% to 2.5% have been validated in some studies (17, 18). Similarly, for invasiveness, radiological invasion is given more credence than histopathological evidence (4, 17). However, a comprehensive evaluation of both radiological and histopathological parameters has been suggested by others, bearing in mind that parasellar invasion may be through natural pathways rather than true invasion (19). Hence, we used the criteria of invasion as either radiological or histopathological in the current study.

The overall response to TMZ in APAs varies between 37% and 60%, depending on the study population, criteria used for response (CD or CD/SD), and radiological or biochemical response (12, 20–22). Discordance between biochemical and radiological response is described, with a higher proportion of patients attaining biochemical than radiologic response (21). We, however, observed a higher radiologic than biochemical response, probably because TMZ was used in some patients for tumor control alone as they already had attained normal hormone levels. The most consistent parameter predicting response in various studies is the functionality of the tumor, with a more significant response to TMZ seen in functional tumors (20, 21, 23). The current study also found higher response rates with functional tumors. Though the most favorable response has been earlier reported in corticotropinomas (22), our study provided evidence that patients with acromegaly may have higher response rates than prolactinomas. The other reported predictor of response to TMZ is the expression of cell-cycle markers, most frequently MGMT, followed by MSH2/MSH6 in some reports (1, 23). However, this has not been confirmed in other studies (24, 25). Lower response of NFPAs to TMZ than functioning adenomas is not explained by differential MGMT expression, as low MGMT has been noted with almost similar frequency in both functional and non-functional tumors (26). In this context, it has been argued that MGMT may not explain the majority of the DNA repair as it is responsible for removing methyl adducts introduced by TMZ which accounts for only 10% of the cell-repair pathways (10). Other involved cell-repair pathways include the MPG pathway, which acts by correcting the base-excision repair (BER), which may account for the remainder of the cell-cycle defects. However, we did note the association of low-to-intermediate MGMT expression with response to TMZ, as has been reported by others (1, 21). We also derived the optimal cutoff of MGMT *h*-score in the adenoma specimen as being significantly associated with the response to TMZ. This simple histopathological parameter may be used for risk stratification and prediction of response to TMZ and has been reported in pancreatic NETs but not hitherto in pituitary adenomas (27). The other novel finding that emerged from the study was the fact that early initiation of TMZ therapy was associated with higher rates of response. Various therapies have been tried or targeted in aggressive or recurrent APAs with varying results (28–31). However, early TMZ use in the management algorithm of APAs has not been previously reported and merits trial in a larger number of patients as part of the treatment strategy in APAs (32, 33).

Furthermore, multiple surgeries were required in the non-responder group as compared with the responder group, despite similar adenoma volume at baseline. Even after multiple surgeries, these patients demonstrated suboptimal response. This suggests that TMZ is non-inferior, or probably superior to multiple surgeries for patients with APAs. Rather than subjecting patients to multiple surgeries, instituting a trial of TMZ therapy may be helpful, at least in functional tumors with low-to-intermediate MGMT expression.

TMZ was generally well tolerated and a majority of adverse events were mild. Our observation of slightly higher rates of adverse events is probably attributable to the fact that all events, related or unrelated, and irrespective of severity were included (21). Three patients developed cytopenias (all responders), necessitating withdrawal of TMZ in them. Further therapy was offered to these patients, with one of them being initiated on long-acting somatostatin analog (acrogigantism) and the other given a daily low-dose TMZ rechallenge therapy (prolactinoma), but she was lost to follow-up. In the current study, the duration of use of TMZ was 3 months or more in all patients. The most common reasons for discontinuation were cost, intolerance/adverse effects, or satisfactory response to TMZ.

The strengths of the study include homogeneity in management by a common multidisciplinary team and evaluation of efficacy and safety of early initiation of TMZ, especially in the setting of non-corticotroph tumors. We do perceive limitations like small number and the fact that tumor response in these patients may have been due to TMZ alone, prior or concurrently administered radiotherapy, or both. However, in any study of similar nature, this is a challenge to be resolved.

## Conclusion

Patients with functional tumors with low-to-intermediate MGMT immunoexpression were more likely to respond to temozolomide. Early initiation of the drug favored a higher likelihood of response and can be used in patients with an aggressive tumor phenotype.

## Prior Presentation

The paper was partly presented as an abstract at the Society for Endocrinology, British Endocrine Society Conference, 2019, Endocrine Abstracts (2019) 65 P286 | DOI: 10.1530/endoabs.65.P286.

## Data Availability Statement

The original contributions presented in the study are included in the article/**Supplementary Material**. Further inquiries can be directed to the corresponding author.

## Ethics Statement

The studies involving human participants were reviewed and approved by the Institutional Ethics Committee, PGIMER. The patients/participants provided their written informed consent to participate in this study.

## Author Contributions

LD helped in patient management, compiled, analyzed and interpreted the data, and drafted the manuscript. NG helped in data compilation, analysis and interpretation. PD conceptualized the idea, supervised patient management, and edited the manuscript. RW edited the manuscript. KV, KG, and BDR supervised the histopathological evaluation and edited the manuscript. AR and RS helped in performing the immunohistochemical evaluation under the supervision of all 3 senior histopathologists. SS performed hematopathological evaluation and edited the manuscript. CKA provided radiological expertise and edited the manuscript. AB helped in patient management. SSD performed the surgeries and edited the manuscript. MT performed gamma knife radiosurgery and edited the manuscript. All authors have read and approved the final version of the manuscript.

## Conflict of Interest

The authors declare that the research was conducted in the absence of any commercial or financial relationships that could be construed as a potential conflict of interest.

## Publisher’s Note

All claims expressed in this article are solely those of the authors and do not necessarily represent those of their affiliated organizations, or those of the publisher, the editors and the reviewers. Any product that may be evaluated in this article, or claim that may be made by its manufacturer, is not guaranteed or endorsed by the publisher.

## Supplementary Material

Supplementary Figure 1 Schematic representation of the workflow for selection of aggressive pituitary adenomas for temozolomide therapy. TMZ, Temozolomide; NFPA, Non-functioning pituitary adenoma; D2A, Dopamine receptor agonist; SRL, Somatostatin receptor ligand.

Supplementary Figure 2 Graph depicting the hormonal and tumoral response of each individual patient to temozolomide. The first 11 patients were non-responders while the remaining 24 were responders
