# Supplementary figures and images for: Early Initiation of Temozolomide Therapy May Improve Response in Aggressive Pituitary Adenomas
Source: Front Endocrinol (Lausanne). 2021 Dec 17;12:774686. doi: 10.3389/fendo.2021.774686 (PMC8718901; doi:10.3389/fendo.2021.774686)

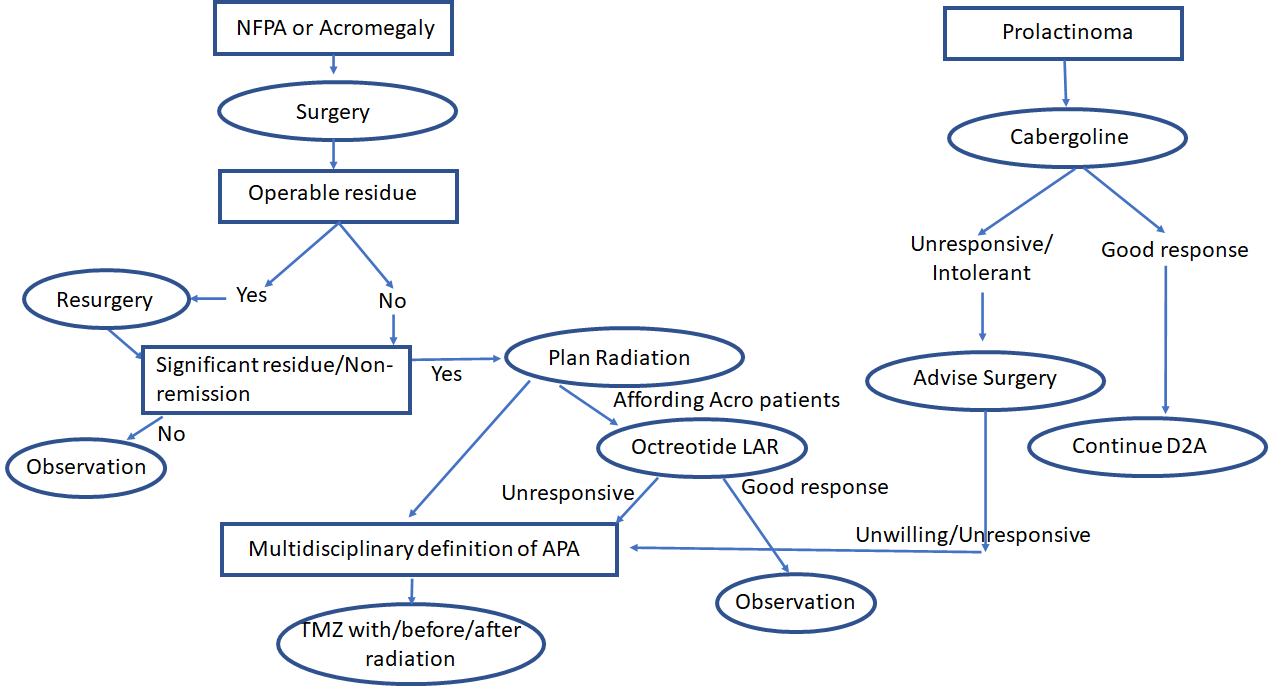

Supplement: Supplementary Figure 1 — Schematic representation of the workflow for selection of aggressive pituitary adenomas for temozolomide therapy. TMZ, Temozolomide; NFPA, Non-functioning pituitary adenoma; D2A, Dopamine receptor agonist; SRL, Somatostatin receptor ligand. [file Image_1.tif]

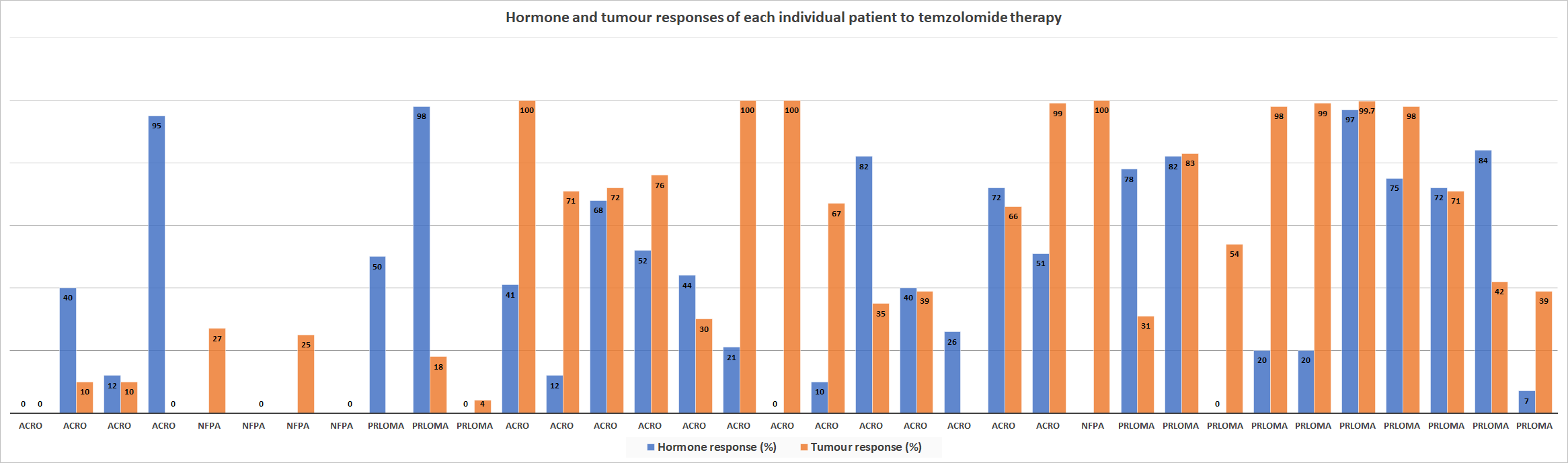

Supplement: Supplementary Figure 2 — Graph depicting the hormonal and tumoral response of each individual patient to temozolomide. The first 11 patients were non-responders while the remaining 24 were responders [file Image_2.tif]
