# Supplementary material for: Early Initiation of Temozolomide Therapy May Improve Response in Aggressive Pituitary Adenomas
Source: Front Endocrinol (Lausanne). 2021 Dec 17;12:774686. doi: 10.3389/fendo.2021.774686 (PMC8718901; doi:10.3389/fendo.2021.774686)
Supplement: Supplementary file 3 [file Table_1.docx]

**Supplementary Table 1: Comparative parameters between responders and non-responders based on the tumour subtype**

| **Acromegaly** | | | |
| --- | --- | --- | --- |
| **Parameter** | **Non-responder** | **Responder** | **p value** |
| Number of patients | 4 | 13 | **-** |
| Age | 38.5 (36.2- 47.5) | 29 (24-38) | 0.14 |
| Gender (%) females | 75 | 69.2 | 0.34 |
| Duration of TMZ use | 10 (3.2-16.0) | 10 (6-16) | 0.57 |
| Hormone response (%) | 26 (3.0-81.2) | 41.0 (16.5-60.0) | 0.65 |
| Tumour response (%) | 5 (0.0-10.0) | 71.5 (45.7-99.5) | 0.001 |
| **Prolactinoma** | | | |
| **Parameter** | **Non-responder** | **Responder** | **p value** |
| Number of patients | 3 | 10 | - |
| Age | 30 (20-40) | 34 (23-46.5) | 0.75 |
| Gender (%) females | 33.3 | 30 | 0.64 |
| Duration of TMZ use | 6 (3-8) | 9.5 (6-12.5) | 0.16 |
| Hormone response (%) | 50 (0-98) | 73.5 (16.7-82.5) | 0.98 |
| Tumour response (%) | 11 (4-18) | 77 (41.25-98.25) | 0.03 |
| **Non-functioning pituitary adenomas** | | | |
| **Parameter** | **Non-responder** | **Responder** | **p value** |
| Proportion of total population | 4 | 1 | - |
| Age | 39.5 (32.75-51.50) | 60 | - |
| Gender (%) females | 25 | 0 | - |
| Duration of TMZ use | 6 (3-15) | 60 | - |
| Hormone response (%) |  |  | - |
| Tumour response (%) | 12.5 (0-26.5) | 100 | - |
