# Supplementary material for: Early Initiation of Temozolomide Therapy May Improve Response in Aggressive Pituitary Adenomas
Source: Front Endocrinol (Lausanne). 2021 Dec 17;12:774686. doi: 10.3389/fendo.2021.774686 (PMC8718901; doi:10.3389/fendo.2021.774686)
Supplement: Supplementary file 4 [file Table_2.docx]

**Supplementary Table 2: Details of hormone and transcription factor immunohistochemistry of the tumours with non-functioning pituitary adenomas**

| **Patient** | **Hormones** | **Transcription factor IHC** |
| --- | --- | --- |
| 1 (non-responder) | All 6 anterior pituitary hormones negative | SF1, Pit1, TPit negative |
| 2 (non-responder) | All 6 anterior pituitary hormones negative | SF1 positive (100%, 3); Pit1, TPit negative |
| 3 (non-responder) | Prl + | SF1 negative |
| 4 (non-responder) | FSH 10%, 2+ | SF1 positive (50%, 3); Pit1, TPit negative |
| 5 (responder) | GH+ | SF1 negative |
